# Molecular Modeling Studies of 4,5-Dihydro-1*H*-pyrazolo[4,3-*h*] quinazoline Derivatives as Potent CDK2/Cyclin A Inhibitors Using 3D-QSAR and Docking

**DOI:** 10.3390/ijms11103705

**Published:** 2010-09-28

**Authors:** Yong Ai, Shao-Teng Wang, Ping-Hua Sun, Fa-Jun Song

**Affiliations:** 1 Laboratory for Natural Product Chemistry, College of Pharmacy, South Central University for Nationalities, 708 Minyuan Road, Wuhan 430074, China; E-Mails: aiyong0508@126.com (Y.A.); wst418638862@sohu.com (S.-T.W.); 2 Guangdong Province Key Laboratory of Pharmacodynamic Constituents of TCM and New Drugs Research, College of Pharmacy, Jinan University, Guangzhou 510632, China; 3 College of Life Science/Key Laboratory for Biotechnology of the State Ethnic Affairs Commission, South Central University for Nationalities, 708 Minyuan Road, Wuhan 430074, China

**Keywords:** CDK2/cyclin A, 3D-QSAR, CoMFA, CoMSIA, docking

## Abstract

CDK2/cyclin A has appeared as an attractive drug targets over the years with diverse therapeutic potentials. A computational strategy based on comparative molecular fields analysis (CoMFA) and comparative molecular similarity indices analysis (CoMSIA) followed by molecular docking studies were performed on a series of 4,5-dihydro-1*H*-pyrazolo[4,3-*h*]quinazoline derivatives as potent CDK2/cyclin A inhibitors. The CoMFA and CoMSIA models, using 38 molecules in the training set, gave *r*^2^_cv_ values of 0.747 and 0.518 and *r*^2^ values of 0.970 and 0.934, respectively. 3D contour maps generated by the CoMFA and CoMSIA models were used to identify the key structural requirements responsible for the biological activity. Molecular docking was applied to explore the binding mode between the ligands and the receptor. The information obtained from molecular modeling studies may be helpful to design novel inhibitors of CDK2/cyclin A with desired activity.

## 1. Introduction

Essentially all physiological processes and a majority of human diseases involve protein phosphorylation. Given the fact that protein phosphorylation is a primary post-translational mechanism applied by cells to regulate enzymes and other proteins in each of the cell cycle transitions, its deregulation has been regarded as the cause or consequence of many maladies [1–3]. CDKs/cyclins, a series of binary protein kinase, show genetic defects in many malignant diseases such as Alzheimer’s [4], Parkinson’s [5], Nieman-Pick’s diseases [6], and ischemia [7] as well as traumatic brain injury [8], when deregulated. CDKs/cyclins exert their effects via activation of host proteins through phosphorylation of key serine or threonine residues by ATP. It was revealed in previous studies that the inhibitors of these CDKs/cyclins were down-regulated in most of the cancer cells [9,10]. A considerable amount of investigations have been carried out to develop inhibitors that target CDK2/cyclin A for treating cancer, and several CDK2/cyclin A inhibitors have been under clinical evaluation [10]. 3D-QSAR and docking approaches have emerged as one of the most powerful tools in ligand based drug design strategies [11,12]. They have been used to develop efficient models for identifying CDK2/cyclin A inhibitors [13,14].

Recently, a series of compounds containing 4,5-dihydro-1*H*-pyrazolo[4,3-*h*]quinazoline that have potent CDK2/cyclin A inhibitory activities were reported by literature [15]. In this paper, molecular modeling studies of these 4,5-dihydro-1*H*-pyrazolo[4,3-*h*]quinazoline derivatives were performed by using 3D-QSAR and docking approaches. 3D-QSAR including comparative molecular field analysis (CoMFA) and comparative molecular similarity indices analysis (CoMSIA) methods were performed to predict the inhibitory activities of these inhibitors, and to provide the regions in space where interactive fields may influence the activity. Meanwhile, a docking study was employed to investigate the protein-ligand interactions. The constructed models can help not only in understanding the structure-activity relationship of these compounds but can also serve as a useful guide for the design of new inhibitors with desired potencies.

## 2. Results and Discussion

### 2.1. CoMFA Model

**Figure 1:**
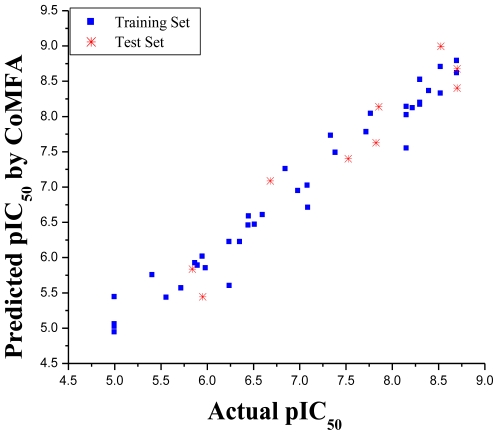
Graph of actual *versus* predicted pIC_50_ values of the training set and the test set molecules using the CoMFA model.

The statistical parameters corresponding to the CoMFA model are listed in Table 1. The CoMFA model of a series of 4,5-dihydro-1*H*-pyrazolo[4,3-*h*]quinazoline derivatives was generated using leave-one-out PLS analysis with an optimized component of 5 to give a good cross-validated correlation coefficient (*r*^2^_cv_) of 0.747 (>0.5), which suggesting that the model should be a reasonable tool for predicting the IC_50_ values. A high non-cross-validated correlation coefficient (*r*^2^) of 0.970 with a low standard error estimate (SEE) of 0.225 was obtained as well as an *F* value of 206.080 and predictive correlation coefficient (*r*^2^_pred_) of 0.942. Contributions of steric and electrostatic fields were 0.599 and 0.401, respectively. The actual and predicted pIC_50_ values of the training set and test set by the model are listed in Table 2, and the graph of actual activity *versus* predicted pIC_50_ of the training set and test set is illustrated in Figure 1.

### 2.2. CoMSIA Model

The statistical parameters corresponding to the CoMSIA model are listed in Table 1. The CoMSIA model, consisting of steric (S), electrostatic (E), hydrophobic (H), hydrogen bond donor (D) and acceptor (A) fields, can be generated using these fields in different combinations. The results of CoMSIA analysis with different combinations are summarized in Table 3. Among the combination models, steric, electrostatic and hydrogen bond acceptor fields played essential roles for the present series of compounds. To confirm whether the addition of hydrophobic, hydrogen bond donor and acceptor fields affect the model, each descriptor was considered along with steric and electrostatic descriptors for generating the model. Inclusion of the hydrophobic field descriptor caused a reduction in both *r*^2^_cv_ and *r*^2^_pred_, which implied that the hydrophobic field descriptor may not be crucial for these molecules. Moreover, the removal of steric and electrostatic descriptors (H + D + A) resulted in significant reduction in *r*^2^_cv_, *r*^2^ and *r*^2^_pred_. The S + E + A combination was better than the S + E + H + D + A combination in every statistical parameter, which suggested that the steric, electrostatic and hydrogen bond acceptor functional groups were of extreme significance for the inhibitory activity. In conclusion, the combination of steric, electrostatic and hydrogen bond acceptor fields was selected as the best model.

**Figure 2:**
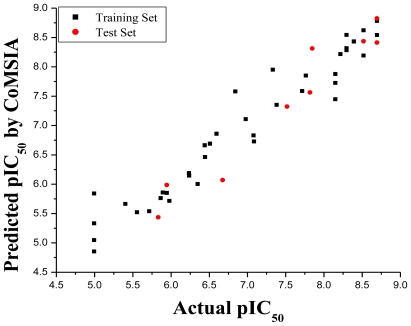
Graph of actual *versus* predicted pIC_50_ values of the training set and the test set molecules using the CoMSIA model.

The CoMSIA model with a combination of steric, electrostatic and hydrogen acceptor fields gave a good cross-validated correlation coefficient (*r*^2^_cv_) of 0.518 (>0.5) with an optimized component of 6. A high non-cross-validated correlation coefficient (*r*^2^) of 0.934 was attained, as well as a low standard error estimate (SEE) of 0.339, *F* value of 72.528 and predictive correlation coefficient (*r*^2^_pred_) of 0.931. Contributions of steric, electrostatic and hydrogen bond acceptor fields were 0.373, 0.472 and 0.155, respectively. The actual and predicted pIC_50_ values and residual values for the training set and test set compounds are listed in Table 2. The association between actual and predicted pIC_50_ of the training set and test set compounds is illustrated in Figure 2.

### 2.3. CoMFA Contour Maps

**Figure 3:**
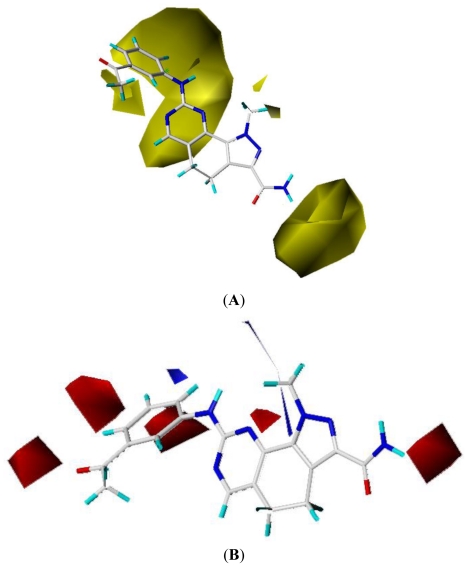
Std* coeff contour maps of CoMFA analysis with 2 Å grid spacing in combination with compound 19: (**A**) Steric fields: green contours indicate regions where bulky groups increase activity; yellow contours indicate regions where bulky groups decrease activity, and (**B**) Electrostatic fields: blue contours (80% contribution) represent regions where electron-donating groups increase activity; red contours (20% contribution) represent regions where electron-withdrawing groups increase activity.

To view the information of the resultant 3D-QSAR model, CoMFA contour maps were generated to rationalize the regions in 3D space around the molecules where changes in the steric and electrostatic fields were predicted to enhance or lessen the activity of the compound. The CoMFA steric and electrostatic contour maps are shown in Figure 3.

The steric field is characterized by green and yellow contours, in which yellow contours indicate regions where minor groups would be favorable, while the green contours represent regions where minor groups would decrease the activity. Compound 19 was selected as a reference structure. As shown in Figure 3A, the N-1 position (R_1_) was surrounded by two small yellow contours, which suggested a minor group at this position would increase the inhibitory potency. This may explain why compounds 01, 02, 04 which possessed a minor group (e.g., Me, H) at R_1_ showed significantly increased activities compared to those with a bulky substituent. For instance, compounds 1–8 had an order for the potency of 01 > 02 > 05 > 03 > 08 > 07, with the corresponding R_1_ substituent Me, F_3_CCH_2_-, Cyclohexane, Phenyl, 1-piperidine-CH_2_-CH_2_-, 1-methyl-piperidine-, respectively. The presence of the yellow contour around the C-3 (R_2_) position also suggested a bulky group at this region would be unfavorable. By checking up all the C-3 modified compounds, it was found that derivatives 1 and 9–14 have the activity order of 1 (R_2_ = NH_2_) > 10 (R_2_ = OH) > 11 (R_2_ = NHMe) > 9 (R_2_ = OEt) > 12 (R_2_ = NHcyclopropyl) > 13 (R_2_ = NHcyclopentyl) > 14 (R_2_ = NHPh). This is satisfactory in accordance with the contour map. The large yellow contour around the benzene at R_3_ indicated that minor groups at this position may benefit potency. This may explain why compound 28 (R_3_ = SMe) was more potential than 34 (R_3_ = SO_2_NH_2_), while compound 34 (R_3_ = SO_2_NH_2_) was more active than 40 (R_3_ = SPh). Comparing compound 27 (R_3_ = Me) with 31 (R_3_ = *i*-Pr), as well as 18 (R_3_ = Ac) with 32 (R_7_ = CO_2_Me), it could be easily found that their activity discrepancies can also be explained by this yellow contour.

The electrostatic field (Figure 3B) is indicated by blue and red contours, which exhibit the regions where electron-donating groups and electron-withdrawing groups would be favorable, respectively. Compound 19 was selected as a reference molecule again. In the CoMFA electrostatic field, a strip blue contour around the N-1 (R_1_) side chain revealed the electron-donating substituent was essential for the inhibitory activity. Take the compound 2 (R_1_ = CF_3_CH_2_) for an example: The strong electron-withdrawing -CF_3_ group at the terminal of N-1 side chain in compound 2 resulted in significantly decreased activity compared to the compound 1 with the electron-donating substituent -CH_3_. The red contour near the C-3 (R_2_) position demonstrated that the electron-withdrawing groups at this position would benefit potency, this may be the reason why compounds 9, and 11–13, which possessed electron-donating groups, had decreased potencies compared to the compounds with -OH group such as compound 10 (R_2_ = OH). The three red contours around the benzene at R_3_ revealed that the electron-withdrawing groups at this position may increase the potency. For instance, compounds 30, 29, 31 had an order for the activity of 30 > 29 > 31, with the corresponding R_3_ substituent -F, -NHMe, *i*-Pr, respectively.

### 2.4. CoMSIA Contour Maps

**Figure 4:**
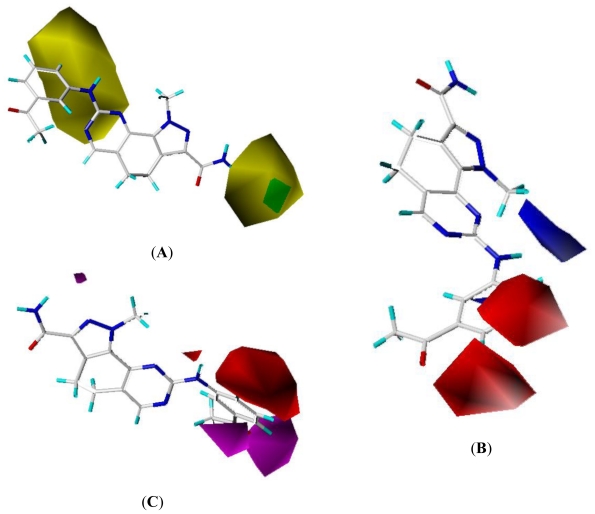
Std* coeff contour maps of CoMSIA analysis with 2 Å grid spacing in combination with compound 19: (**A**) Steric fields: Green contours (80% contribution) indicate regions where bulky groups increase activity; yellow contours (20% contribution) indicate regions where bulky groups decrease activity; (**B**) Electrostatic fields: Blue contours (80% contribution) represent regions where electron-donating groups increase activity; red contours (20% contribution) represent regions where electron-withdrawing groups increase activity; (**C**) hydrogen bond acceptor contour map. The magenta and red (80% and 20% contributions) contours indicate favorable and unfavorable hydrogen bond acceptor groups.

The best combination model for CoMSIA were steric, electrostatic and hydrogen bond acceptor fields (Table 3). The hydrophobic and hydrogen bond donor fields were not essential for the CoMSIA model, thus their contours were not generated. The CoMSIA steric and electrostatic field contour maps were approximately similar to the corresponding CoMFA contour maps, therefore the figures were not illustrated, either (Figure 4A or 4B).

The hydrogen bond acceptor field contour map of CoMSIA is shown in Figure 4 using compound 19 as a reference molecule. The magenta and red contours represent favorable and unfavorable hydrogen bond acceptor groups. In the CoMSIA hydrogen bond fields, the magenta contour near the benzene (*m*,*p*-R_3_) revealed that hydrogen bond acceptor groups may benefit the potency. The -F, -O, and -N atom at this position acted as hydrogen bond acceptor, this may explain why compounds 16–17, 19–20, 22–23 and 25–26 showed relatively better activities. One huge red contour around the benzene (*o*-R_3_) revealed that hydrogen bond acceptor groups may decrease the inhibitory activity. For example, compounds 1, 15, 29, 30 had an order for the activity of 1 > 30 > 29 > 15, with the corresponding *o*-R_3_ substituent -H, -F, -NHMe, -CF_3_, respectively.

### 2.5. Docking Analysis

Docking was implemented to find the probable binding conformations between these 4,5-dihydro-1*H*-pyrazolo[4,3-*h*]quinazoline derivatives and the receptor, furthermore, to check the reliability of the 3D-QSAR models established. Since the crystal structure of CDK2/cyclin A was known, we docked compound 19 into the allosteric site of CDK2/cyclin A (PDB code 2WXV), and the surfex-dock total score was 9.17.

**Figure 5:**
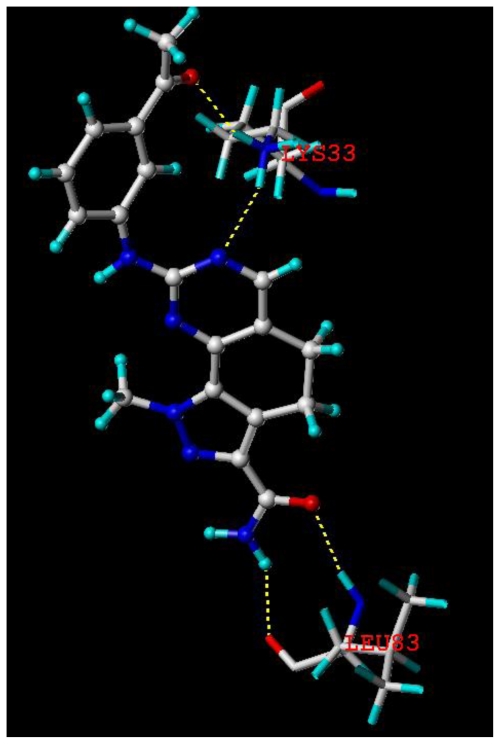
The binding mode between compound 19 and the allosteric site of CDK2/cyclin A (PDB code 2WXV). Key residues and hydrogen bonds are labeled.

As shown in Figure 5, the key residues and hydrogen bonds were labeled, namely: the O and N at the C-3 position of the pyrazolo ring in compound 19 served as hydrogen bond acceptor and donor by forming two H-bond with the -NH_2_, -OH group of LEU83 residue, respectively. The N atom of the pyrimidine ring and the O atom of m-Ac in compound 19 acted as the hydrogen bond acceptors by forming two H-bonds with -NH_2_ group of LYS33 residue. The results confirmed the observation from the CoMSIA hydrogen bond acceptor contour map.

**Figure 6:**
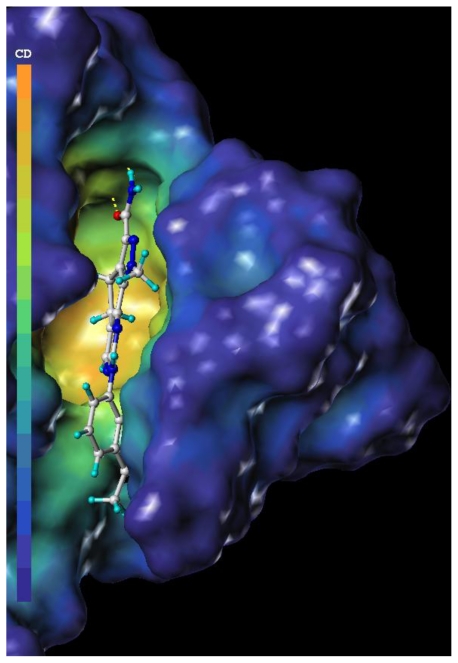
MOLCAD cavity depth potential surface of the allosteric site of CDK2/cyclin A (PDB code 2WXV) within the compound 19. Light red color denotes the deepest depth.

In order to test and verify the use of docking, the MOLCAD surface with cavity depth potential was generated and is shown in Figure 6. The cavity depth measures how deep a surface point is located inside a cavity of a molecule. The cavity depth color ramp ranges from blue (low depth values represent outside of the molecule) to light red (high depth values represent cavities deep inside the molecule). It can be observed that the whole molecule was in the light red region (Figure 6) which revealed that compound 19 was placed well in the allosteric site.

**Figure 7:**
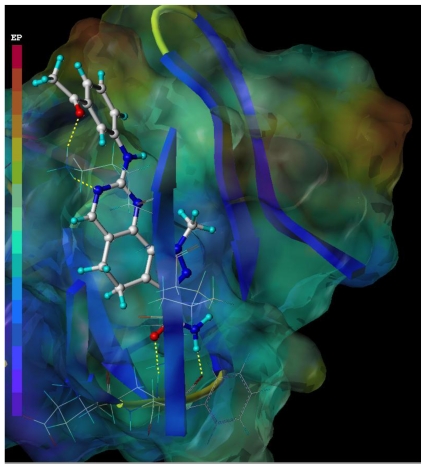
The MOLCAD electrostatic potential surface of the allosteric site of CDK2/cyclin A (PDB code 2WXV) within the compound 19. The color ramp for EP (electrostatic potential) ranges from red (most positive) to purple (most negative).

The MOLCAD surface of the allosteric site was developed and displayed with electrostatic potential to test and verify the CoMFA electrostatic contour map (Figure 7). The molecular electrostatic potential on a protein surface can be applied to find the sites that act attractively on ligands by matching opposite colors. The compound 19 was docked into the allosteric site; the red color shows the electron-withdrawing zone and purple color shows electron-donating zone. The observation seen in Figure 7 was satisfactory according to that of CoMFA electrostatic contour map. In detail, the R_2_ region is in the red zone, which suggested that electron-withdrawing substituent would be favorable; the R_1_ region is in a blue zone, which indicated that electron-donating groups may be favorable.

**Figure 8:**
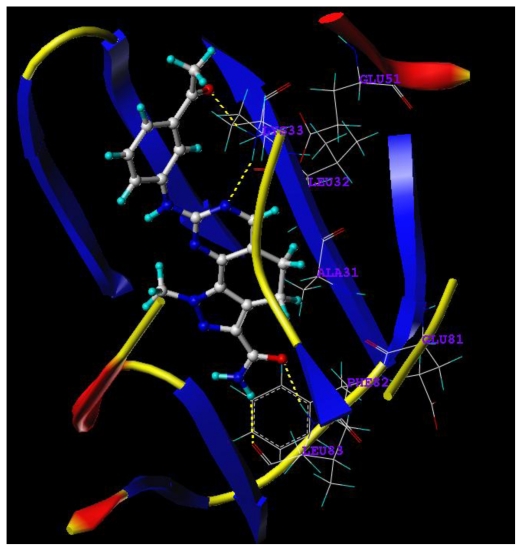
The MOLCAD Ribbon Surfaces of the allosteric site of CDK2/cyclin A (PDB code 2WXV) within the compound 19.

To better visualize the protein structure, in this paper, protein residues were explored using the ribbon program (Figure 8). The protein backbone is drawn as a ribbon or tube. Representations of proteins in Richardson style use arrows for beta strands, cylinders for alpha helices and tubes for coils and turns. As showed in Figure 8, the two protein residues involved—LYS33 and LEU83—lie within arrows designating beta strands.

### 2.6. Design of New Molecules Based on COMFA, CoMSIA and Docking Studies

**Figure 9:**
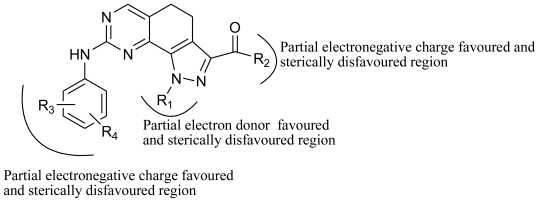
Structural requirements for binding and inhibitory activity of inhibitors.

**Figure 10:**
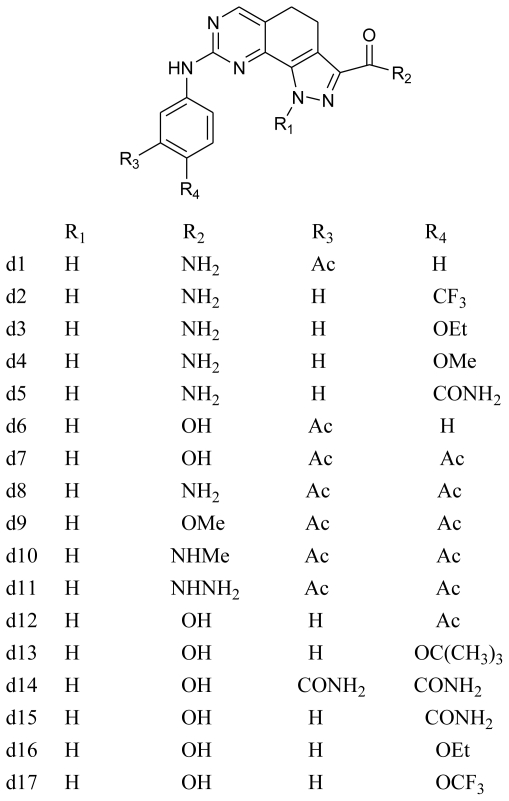
Structure of newly designed molecules.

**Figure 11:**
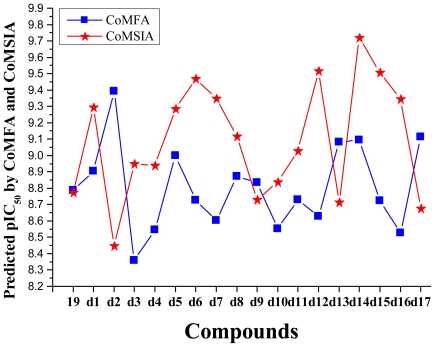
Graph of the predicted pIC_50_ of the newly designed molecules *versus* compound 19.

The detailed contour map analysis of both COMFA and CoMSIA models and the docking analysis empowered us to identify structural requirements for the observed inhibitory activity (Figure 9). The molecules were modified to further improve the inhibition activity toward CDK2/CyclinA. Compound 19 were chosen as a reference structure to design new molecules to obtain a number of new potent molecules (Figure 10). The newly designed molecules were docked into the protein active site. The COMFA and CoMSIA models established above were used to predict the activity by applying the 3D-QSAR model. The new molecules showed better dock score and predicted activity (Table 4). The comparison of the predicted activity of the newly designed molecules between CoMFA and CoMSIA models are showed in Figure 11. The designed molecules showed better activity than the reference molecules, which indicates that the 3D-QSAR model has a good predictability and can be used to design new molecules with better activity.

## 3. Materials and Methods

### 3.1. Data Sets

The 47 compounds involved in this study were taken from the literature [15]. The inhibitory activities were reported as IC_50_ against CDK2/cyclin A. The IC_50_ values were converted into pIC_50_ by taking Log (1/IC_50_). The entire derivatives were divided into a training set of 38 compounds and a test set of nine compounds for model validation. The test set compounds were selected randomly. Chemical structures and associated inhibitory activities are shown in Table 5 and Table 1.

### 3.2. Molecular Modeling and Alignment

**Figure 12:**
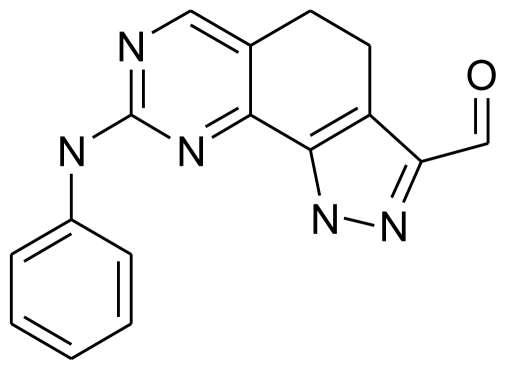
Common substructure used for alignment.

**Figure 13:**
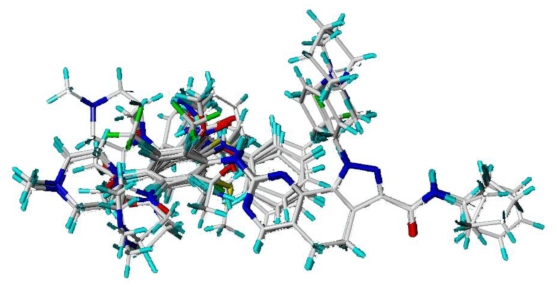
Alignment of the compounds used in the training set.

Molecular modeling and statistical analysis were performed using the molecular modeling package SYBYL 8.1 Tripos, Inc. [16]. The three-dimensional structures of all compounds were constructed using the Sketch Molecule module. Energy minimization of each structure was performed using the SYBYL energy minimizer Tripos force field and Gasteiger-Hückel charge [17,18]. All of the compounds were aligned into a lattice box by fitting with common substructure (Figure 12) using compound 19 as a template, which was one of the most active compounds. The conformation of the template was based on crystallographic ligand/receptor complex. (The aligned molecules in the training set are shown in Figure 13).

### 3.3. CoMFA and CoMSIA Modeling

The CoMFA descriptor fields including the steric fields and the electrostatic fields were calculated at each lattice with grid spacing of 1 Å and extending to 4 Å units in all three dimensions within defined region [17,18]. The Van Dar Waals potentials and Coulombic terms, which represented steric and electrostatic fields, respectively, were calculated by using the standard Tripos force field. In CoMFA method, a sp^3^ hybridized carbon atom with a charge of 1e was used as a probe atom, the energy values of the steric and electrostatic fields were truncated at 30 kcal/mol [18–20].

The steric, electrostatic, hydrophobic, hydrogen bond donor and hydrogen bond acceptor CoMSIA potential fields were calculated at each lattice intersection of a regularly spaced grid of 1 Å and extending to 4 Å using a probe atom with radius 1.0 Å, +1.0 charge, and hydrophobic and hydrogen bond properties of +1. The attenuation factor was set to the default value of 0.3 [21].

### 3.4. Partial Least Squares (PLS) Analysis

The partial least-squares (PLS), an extension of multiple regression analysis, was used to linearly correlate the CoMFA and CoMSIA fields to the pIC_50_ values. The cross-validation analysis was performed using the leave-one-out (LOO) method in which one molecule was removed from the data set and its activity was predicted using the model derived from the rest of the data set [20]. PLS was used in conjunction with the cross-validation option to determine the optimum number of components (ONC) which were then used in deriving the final CoMFA and CoMSIA model without cross-validation. The ONC was the number of components resulted in highest cross-validated correlated correlation coefficient (*r*^2^_cv_) [20–22]. Column filtering was used at the default value of 2.0 kcal/mol in the cross-validation part. The final models were developed with ONC by using non-cross-validated analysis equal yielded the highest correlation coefficient (*r*^2^) [23].

### 3.5. Predictive Correlation Co-Efficient (r^2^_pred_)

The predictive abilities of 3D-QSAR models were validated by predicting the activities of a test set of eight compounds that were not included in the training set. These molecules were aligned to the template and their pIC_50_ values were predicted by the produced models which were obtained using the training set. The predictive correlation coefficient (*r*^2^_pred_), based on the molecules of test set, was calculated using Equation (1):

(1) $${r^{2}}_{\text{pred}}=(\text{SD}-\text{PRESS})/\text{SD}$$

In this equation, SD is the sum of the squared deviations between the inhibitory activities of the test set and the mean activity of the training molecules and PRESS is the sum of squared deviations between predicted and actual activity values for each molecule in the test set [22–26].

### 3.6. Molecular Docking

To study the protein-ligand interactions, compound 19 with a high pIC_50_ value was selected as a reference compound and docked into the ATP-binding site of CDK2/cyclin A. The ATP binding site was situated in a deep cleft between a amino-terminal lobe (residues 1–85) and a carboxy-terminal lobe which contains the catalytic residues conserved among eukaryotic protein kinases. The ATP pocket of CDK2/cyclin A has an impressive capacity to accommodate a variety of inhibitors containing flat heterocyclic structures [27]. The Surflex-Dock using an empirical scoring function and a patented search engine to dock ligands into a protein’s binding site was used to investigate molecular docking [26]. The scoring function was tuned to predict the binding affinities of protein/ligand complexes, with its output being represented in units of -log(*K*_d_)^2^. The terms, in rough order of significance, were hydrophobic complementarity, polar complementarity, entropic terms, and solvation terms. The full scoring function was the sum of each of these terms [27].

The crystal structure of CDK2/cyclin A was obtained from Protein Data Bank, having a PDB entry of 2WXV. The CDK2/cyclin A structure was exploited in subsequent docking experiments without energy minimization. The compound 19 was docked into corresponding protein’s binding site by an empirical scoring function and a patented search engine in Surflex-Dock [16]. The automatic docking was applied. All the inhibitor and water molecules from crystal structure have been removed and the polar hydrogen atoms were added.

The MOLCAD (Molecular Computer Aided Design) program was applied to visualize the binding mode between the ligand and protein. MOLCAD calculates and displays the surfaces of channels, Ribbon, and cavities, as well as the separating surface between protein subunits [16]. MOLCAD program provides several types to create a molecular surface, the fast Connolly method which uses a marching cube algorithm to generate the surface was utilized. Other parameters were established by default in software.

## 4. Conclusion

We have employed 3D-QSAR and docking methods to explore the structure-activity relationship of a series of 4,5-dihydro-1*H*-pyrazolo[4,3-*h*]quinazoline derivatives as potent CDK2/cyclin A inhibitors. The CoMFA analysis was used to build statistically significant models with good correlative and predictive capability for the inhibition of CDK2/cyclin A by 47 4,5-dihydro-1*H*-pyrazolo[4,3-*h*] quinazoline derivatives. These models could be used to predict the inhibitory potencies of related structures. The analysis of contours for the CoMFA models has provided a clue about the structural requirement for the observed biological activity for the respective kinases: A more electron-withdrawing group and less bulky substitution on the pyrazolo ring are expected to improve the inhibitory potency. Furthermore, the CoMSIA contour maps along with the docking results offered enough information that more hydrogen bond acceptor groups on the benzene ring (*m*,*p*-R_3_) and more hydrogen bond donor groups on the benzene ring (*o*-R_3_) may benefit the potency. The clues obtained from 3D-QSAR and docking studies can be served as a useful guideline for the amplification of the known CDK2/cyclin A family of inhibitors. The designed molecules based on those parameters showed better activity than the reference molecules, which indicates that the 3D-QSAR model that was generated has a good predictability and can be used to design new molecules with better activity. These molecules can be synthesized to generate a greater number of molecules with required pharmacokinetics for further clinical studies.

## Figures and Tables

**Table 1:** Results of CoMFA and CoMSIA Models.

PLS Statistics	CoMFA	CoMSIA
*r*^2^_cv_^a^	0.747	0.518
*r*^2^^b^	0.970	0.934
ONC^c^	5	6
SEE^d^	0.225	0.339
*F* value^e^	206.080	72.528
*r*^2^_pred_^f^	0.942	0.931
Field contribution		
Steric	0.599	0.373
Electrostatic	0.401	0.472
Hydrophobic	-	-
H-bond Donor	-	-
H-bond Acceptor	-	0.155

a cross-validated correlation coefficient;

b non-cros-validated coefficient;

c optimal number of components;

d standard error of estimate;

e value F-test value;

f predictive correlation coefficient.

**Table 2:** The actual pIC_50_s, predicted pIC_50_s (Pred.) and their residuals (Res.) of the training and test set molecules.

Compd. No.	pIC_50_	CoMFA		CoMSIA	

	Actual	Pred.	Res.	Pred.	Res.
1*	8.699	8.402	0.297	8.405	0.294
2	8.301	8.166	0.135	8.535	−0.234
3*	7.824	7.629	0.195	7.555	0.269
4	8.699	8.613	0.086	8.534	0.165
5	8.155	7.547	0.608	7.440	0.715
6	7.337	7.727	−0.390	7.943	−0.606
7	6.983	6.942	0.041	7.101	−0.118
8*	7.523	7.402	0.121	7.315	0.208
9	6.600	6.602	−0.002	6.854	−0.254
10	8.523	8.701	−0.178	8.615	−0.092
11	7.721	7.778	−0.057	7.581	0.140
12	7.092	6.704	0.388	6.720	0.372
13	5.409	5.750	−0.341	5.658	−0.249
14	6.241	6.219	0.022	6.183	0.058
15	6.514	6.466	0.048	6.684	−0.170
16	8.222	8.115	0.107	8.210	0.012
17*	8.523	8.994	−0.471	8.428	0.095
18	5.899	5.887	0.012	5.851	0.048
19	8.699	8.788	−0.089	8.774	−0.075
20*	8.699	8.676	0.023	8.819	−0.120
21	7.387	7.485	−0.098	7.345	0.042
22	8.301	8.521	−0.220	8.275	0.026
23	8.398	8.360	0.038	8.424	−0.026
24*	6.680	7.088	−0.408	6.063	0.617
25	8.301	8.194	0.107	8.310	−0.009
26*	7.854	8.137	−0.283	8.305	−0.451
27	8.523	8.324	0.199	8.185	0.338
28	6.848	7.253	−0.405	7.573	−0.725
29	7.770	8.037	−0.267	7.844	−0.074
30	8.155	8.136	0.019	7.719	0.436
31	6.446	6.455	−0.009	6.656	−0.210
32*	5.839	5.837	0.002	5.429	0.410
33	5.561	5.430	0.131	5.515	0.046
34	5.951	6.011	−0.060	5.844	0.107
35	5.871	5.920	−0.049	5.754	0.117
36	5.721	5.564	0.157	5.532	0.189
37	6.243	5.598	0.645	6.139	0.104
38	6.355	6.220	0.135	5.995	0.360
39	5.000	5.018	−0.018	4.846	0.154
40	5.000	5.439	−0.439	5.833	−0.833
41	8.155	8.019	0.136	7.869	0.286
42	5.984	5.849	0.135	5.709	0.275
43*	5.950	5.443	0.507	5.979	−0.029
44	5.000	4.940	0.060	5.326	−0.326
45	5.000	5.053	−0.053	5.040	−0.040
46	7.086	7.019	0.067	6.823	0.263
47	6.450	6.583	−0.133	6.456	−0.006

* Test Set Molecules.

**Table 3:** Summary of CoMSIA Analysis.

	*r*^2^_cv_	*r*^2^	ONC	SEE	*F* value	*r*^2^_pred_
S + E	0.593	0.943	6	0.315	85.009	0.965
S + E + H	0.415	0.947	6	0.303	92.344	0.887
S + E + D	0.449	0.940	6	0.322	80.894	0.937
S + E + A*	**0.518**	**0.934**	**6**	**0.339**	**72.528**	**0.931**
H + D + A	0.276	0.637	2	0.746	30.677	0.555
S + E + H + D	0.337	0.953	6	0.287	103.677	0.848
S + E + H + A	0.397	0.944	6	0.311	87.122	0.843
S + E + D + A	0.422	0.892	4	0.419	68.166	0.843
S + E + H + D + A	0.355	0.944	6	0.310	87.636	0.769

S: Steric; E: Electrostatic; H: Hydrophobic; D: H-bond Donor; A: H-bond Acceptor;

* Best model for CoMSIA.

**Table 4:** Surflex-Dock total-score and predicted activity of newly designed molecules.

Compound	Predicted pIC_50_		Total-Score
	CoMFA	CoMSIA	
**19**	8.788	8.774	9.17
**d1**	8.903	9.293	8.62
**d2**	9.393	8.447	7.20
**d3**	8.360	8.949	9.02
**d4**	8.547	8.940	6.53
**d5**	8.998	9.286	7.27
**d6**	8.726	9.470	6.57
**d7**	8.603	9.347	8.36
**d8**	8.871	9.116	6.68
**d9**	8.833	8.731	7.13
**d10**	8.552	8.837	6.50
**d11**	8.730	9.027	7.82
**d12**	8.628	9.517	7.51
**d13**	9.082	8.713	5.89
**d14**	9.094	9.719	8.45
**d15**	8.722	9.507	7.30
**d16**	8.527	9.345	9.25
**d17**	9.115	8.675	5.99

**Table 5:** The Structures of the Training and Test Set Molecules.

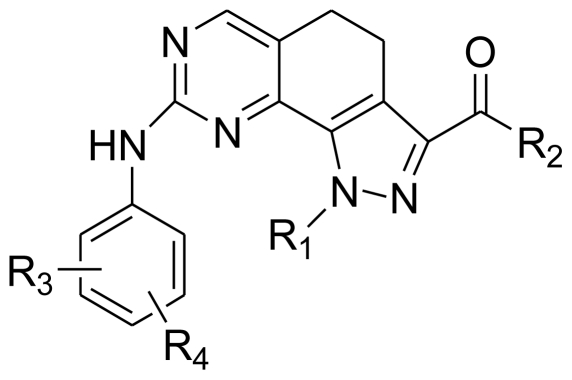				
Compd. No.	Substituent			
	R_1_	R_2_	R_3_	R_4_
1	Me	NH_2_	H	H
2	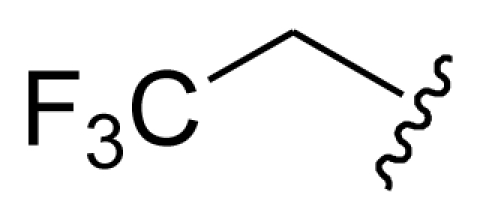	NH_2_	H	H
3	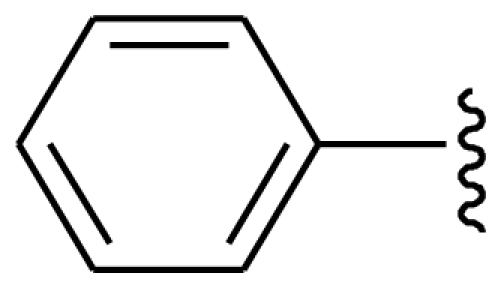	NH_2_	H	H
4	H	NH_2_	H	H
5	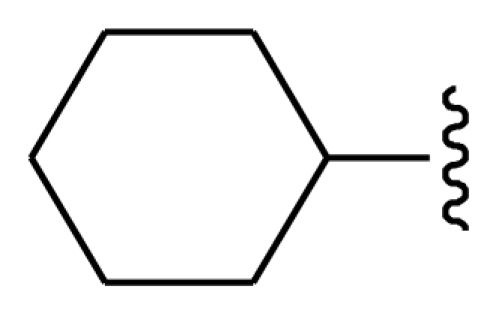	NH_2_	H	H
6	*i*-Pr	NH_2_	H	H
7	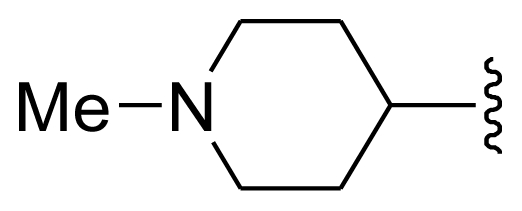	NH_2_	H	H
8	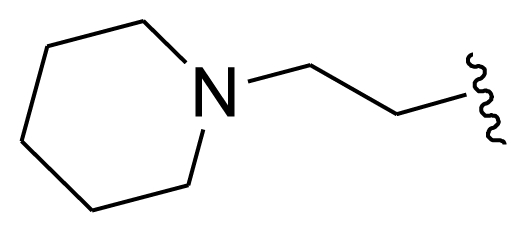	NH_2_	H	H
9	Me	OEt	H	H
10	Me	OH	H	H
11	Me	NHMe	H	H
12	Me	NHcyclopropyl	H	H
13	Me	NHcyclopentyl	H	H
14	Me	NHPh	H	H
15	Me	NH_2_	*o*-CF_3_	H
16	Me	NH_2_	*m*-CF_3_	H
17	Me	NH_2_	*p*-CF_3_	H
18	Me	NH_2_	*o*-Ac	H
19	Me	NH_2_	*m*-Ac	H
20	Me	NH_2_	*p*-Ac	H
21	Me	NH_2_	*o*-OMe	H
22	Me	NH_2_	*m*-OMe	H
23	Me	NH_2_	*p*-OMe	H
24	Me	NH_2_	*o*-NO_2_	H
25	Me	NH_2_	*m*-NO_2_	H
26	Me	NH_2_	*p*-NO_2_	H
27	Me	NH_2_	*o*-Me	H
28	Me	NH_2_	*o*-SMe	H
29	Me	NH_2_	*o*-NHMe	H
30	Me	NH_2_	*o*-F	H
31	Me	NH_2_	*o*- *i-*Pr	H
32	Me	NH_2_	*o*-CO_2_Me	H
33	Me	NH_2_	*o*-CONH_2_	Cl
34	Me	NH_2_	*o*-SO_2_NH_2_	H
35	Me	NH_2_	*o*-Ph	H
36	Me	NH_2_	*o*-OPh	H
37	Me	NH_2_	*o*-benzyl	H
38	Me	NH_2_	*o*-NHPh	H
39	Me	NH_2_	*o*-benzoyl	H
40	Me	NH_2_	*o*-SPh	H
41	Me	NH_2_	*o*-NH_2_	H
42	Me	NH_2_	*o*-NHAc	H
43	Me	NH_2_	*o*-Ac	3′-(4-methyl-piperazin-1-yl)
44	Me	NH_2_	*o*-Ac	4′-(4-methyl-piperazin-1-yl)
45	Me	NH_2_	*o*-Ac	5′-(4-methyl-piperazin-1-yl)
46	Me	NH_2_	*o*-OMe	4′-(4-methyl-piperazin-1-yl)
47	Me	NH_2_	*o*-OMe	5′-(4-methyl-piperazin-1-yl)
